# Obesity-Related Oxidative Stress and Antioxidant Properties of Natural Compounds in the Enteric Nervous System: A Literature Overview

**DOI:** 10.3390/antiox15010083

**Published:** 2026-01-08

**Authors:** Vincenzo Bellitto, Daniele Tomassoni, Ilenia Martinelli, Giulio Nittari, Seyed Khosrow Tayebati

**Affiliations:** 1 School of Biosciences and Veterinary Medicine, University of Camerino, 62032 Camerino, Italy; vincenzo.bellitto@unicam.it (V.B.); daniele.tomassoni@unicam.it (D.T.); 2 School of Pharmacy, University of Camerino, 62032 Camerino, Italy; ilenia.martinelli@unicam.it (I.M.); giulio.nittari@unicam.it (G.N.)

**Keywords:** enteric nervous system, obesity, inflammation, oxidative stress, antioxidants compounds, bioactive compounds

## Abstract

The enteric nervous system (ENS) constitutes a highly organized and intricate neuronal network comprising two principal plexuses: myenteric and submucosal. These plexuses consist of neurons and enteric glial cells (EGCs). Neurons ensure innervation throughout the intestinal wall, whereas EGCs, distributed within the mucosa, contribute to epithelial barrier integrity and modulation of local inflammatory responses. The ENS orchestrates essential gastrointestinal functions, including motility, secretion, absorption, vascular regulation, and immune interactions with gut microbiota. Under physiological conditions, intestinal homeostasis involves moderate generation of reactive oxygen species (ROS) through endogenous processes such as mitochondrial oxidative phosphorylation. Cellular antioxidant systems maintain redox equilibrium; however, excessive ROS production induces oxidative stress, promoting EGCs activation toward a reactive phenotype characterized by pro-inflammatory cytokine release. This disrupts neuron–glia communication, predisposing to enteric neuroinflammation and neurodegeneration. Obesity, associated with hyperglycemia, hyperlipidemia, and micronutrient deficiencies, enhances ROS generation and inflammatory cascades, thereby impairing ENS integrity. Nevertheless, non-pharmacological strategies—including synthetic and natural antioxidants, bioactive dietary compounds, probiotics, and prebiotics—attenuate oxidative and inflammatory damage. This review summarizes preclinical and clinical evidence elucidating the interplay among the ENS, obesity-induced oxidative stress, inflammation, and the modulatory effects of antioxidant interventions.

## 1. Introduction

Enteric neurons and enteric glial cells (EGCs) comprise the enteric nervous system (ENS). They create a complex network that extends throughout the gastrointestinal tract (GIT) with abundant nerve connections. The Auerbach’s (myenteric) and Meissner’s (submucosal) are two components in this neuroglial network; the first is located within the smooth muscle tissue of the GIT, while the second is in the submucosal tunica, ensuring innervations across all layers of the GIT. The EGCs are also found in the mucosal layer and can regulate the epithelial intestinal barrier permeability and the mucosal interconnection with the immune system. The composition of the enteric neurons is varied, and the function can be differentiated by either their chemical coding or function [1]. Functional classification entails intrinsic primary afferent neurons, interneurons (ascending and descending), as well as motor, secretomotor, and vasomotor neurons, all of which carry the initial reflexes to their muscle innervation. According to the functional definition, chemical coding refers to specific properties of enteric neurons. Some examples are the excitatory motor neurons like cholinergic neurons with acetylcholine (ACh) as their main neurotransmitter, and inhibitory motor neurons such as nitrergic and vasoactive intestinal peptide (VIP)-ergic neurons where nitric oxide (NO) and VIP act as the respective key neurotransmitters [2,3]. EGCs are involved in finely regulated interfaces with enteric neurons and represent the most abundant cell type in the ENS. Recently, they have been shown to have more functions than being the sole neuronal support cells. Multiple studies have demonstrated that EGCs not only regulate intestinal neurotransmission, neurogenesis, and neuronal survival, but also play an important role in intestinal homeostasis and functional and immune command control [4,5,6,7,8]. Crosstalk between enteric neurons and EGCs was previously thought to involve multiple molecular mechanisms. EGCs can upregulate the availability of neurotransmitters, such as NO levels, and, in some cases, prevent neuronal death by releasing reduced glutathione (GSH) [9,10,11]. On the other hand, it has been suggested that neurotransmitters released by enteric neurons, such as ACh and serotonin, work on EGCs’ excitability and may influence motor reflex coordination [5,12]. Additionally, purines released from neurons can activate EGCs due to the presence of purinergic receptors on the cell membrane [13,14]. All these complex interactions maintain the normal function of the ENS to perform the physiological functions associated with these cells. The role played by the ENS in gastrointestinal physiology includes motility, immune profile, secretion and absorption, interaction with the intestinal microbiota, and blood flow control [2]. The ENS, responsible for managing various functions, plays a vital role in maintaining the balance of GIT through a complex interaction between neuroimmune and neuroendocrine systems. Enteric neurons possess receptors for multiple gut hormones, including cholecystokinin, glucose-dependent insulinotropic polypeptide, ghrelin, leptin, glucagon-like peptide 1 (GLP-1), and peptide YY. The coordination between the ENS and these gut hormones, released by enteroendocrine cells, influences the patterns of movement, secretion, and absorption in the GIT. Besides, in conjunction with signals from the central nervous system (CNS), this integration regulates appetite and feeding behavior [15]. Direct interaction between the neuroimmune systems plays a crucial role in safeguarding the gut environment. Molecular mechanisms associated with innate immunity activation are closely linked to enteric neural cells. Immune cells in the local area possess neurotransmitter receptors, while EGCs express Toll-like receptors, major histocompatibility complexes I and II. Furthermore, both enteric neurons and EGCs contribute to activating intestinal macrophages [8,16,17,18]. Compounding this situation, the ENS and gut microbiota are in constant communication. Commensal bacteria can modulate intestinal signaling and EGCs turnover and their differentiation, neuronal excitatory properties, and neurotransmitter release [19,20,21]. Over the past few years, there has been a growing body of evidence indicating that various diseases, including inflammatory bowel diseases (IBDs) and neurodegenerative disorders, can impact the functioning of the ENS. This impact primarily affects gut motility and causes local inflammation. While the exact mechanisms behind this effect on the ENS are not fully understood, it is believed to involve abnormal communication between enteric neurons and EGCs. Inflammation triggers a pathological response in EGCs, known as reactive gliosis or glial reactivity, which can lead to neuronal death. Besides, oxidative stress could work as a predisposing factor, affecting neuronal survival and glial modulation [22,23,24,25].

### 1.1. Search Strategy to Review the Literature

Original articles were searched from different sources on Medline via PubMed, Google Scholar and Scopus using different combinations of the following terms: “enteric nervous system,” “enteric neural network,” “enteric glial cells,” “neuroglial network,” “obesity-related oxidative stress,” “obesity-related inflammation,” “bioactive compounds on gut homeostasis” and “Effects of antioxidant compounds on enteric neuromodulation.” Original papers and the references of the selected articles that met the inclusion criteria, such as preclinical and clinical studies, application of an obesity-promoting dietary plan, evaluation of neuromodulation of the enteric neural network, supplementation with antioxidant compounds, and evaluation of their effects on intestinal neuronal homeostasis, were analyzed. This is in line with the objectives of this review, namely, to better clarify the interconnections between these aspects and provide food for thought for future investigations aimed at achieving clinical use of the discussed natural antioxidant compounds.

### 1.2. Oxidative Stress

Oxygen reactivity is involved in energy production to keep our highly evolved living organisms alive. Although these oxidative functions help the functioning of cellular metabolism as oxidative phosphorylation, they also compromise cell structures that require antioxidant defense systems to survive [26,27]. The regulation of multiple cell processes is ultimately dependent on oxygen and the reactive oxygen species (ROS) derivatives, in which redox balance plays a significant part [28,29]. ROS mainly comprised two byproducts of cellular metabolism: superoxide (O_2_^•−^) and hydrogen peroxide (H_2_O_2_). While ROS and reactive nitrogen species (RNS) are commonly known to be leading causes of cell damage, they also play roles in vital biological activities. These physiological functions include apoptosis, intracellular signaling cascades, transcriptional processes, oxygen sensing, proliferation and differentiation of enterocytes, gut microbiota homeostasis, and smooth muscle tone [30,31,32]. The appropriate levels of ROS and RNS function as mediators. Between RNS, NO is associated with various biological processes [27]. Especially in the GIT environment, NO is used as a neurotransmitter by enteric neurons to enhance the muscular relaxation of smooth muscles necessary for the evacuation of luminal content of the digestive tract. The production of NO derives from the oxidation of L-arginine by three types of nitric oxide synthases (NOS): neuronal NO synthases (nNOS), endothelial NO synthases (eNOS), and inducible NO synthases (iNOS). Only iNOS generates enough concentrations of NO sufficient to provoke cellular damage [33]. Disturbance of this fragile redox balance results in an oxidative environment with an accumulation of ROS and RNS, which promotes changes in lipids, proteins, and DNA that result in cellular damage and inflammation [34,35].

### 1.3. Inflammation

Oxidative stress serves as a signal for the immune system to carry out its normal function. Free radicals are also deployed in response, through a mechanism that works on a small scale to destroy both bacteria and damaged or unhealthy cells, as well as modify them. The response is by changing gene expression and altering protein function. However, free radical signaling is aspecific, so it can cause tissue damage reciprocally triggered by inflammation [32]. Oxidative stress acts as a crucial amplifier of inflammation, as ROS can directly upregulate numerous genes, including those related to the inflammatory response. In addition, oxidative stress increases mucosal permeability, a condition triggered by enterotoxins and antigenic insults that sustain intestinal inflammation. Notably, oxidative stress is also known to stimulate immune activation and may represent a key factor in the development of chronic intestinal inflammation [36,37]. Immune cells relate to enteric neurons and EGCs because they express similar receptors against molecules and neurotransmitters that control the ENS and immune systems, eliciting an inflammatory response. This is enhanced in gut inflammation, which causes structural and functional changes in gut effects [38,39,40,41]. Mucosal inflammation can promote the hyperpolarization of neurons that travel into the mucosa, making them hyperexcitable. Similarly, the ascending neurons look to have an augmented excitability in response to inflammation. Ultimately, colonic interneurons responsible for the final intestinal peristaltic reflex appear to have an increased potential of synaptic communication in the submucosal and myenteric plexus in the inflamed colon [42,43,44,45,46,47,48,49]. In addition, immune cells produce neurotransmitters and neuromodulators such as serotonin and ACh, as well as pro-inflammatory cytokines like tumor necrosis factor-alpha (TNF-α), interleukin-1β (IL-1β) and interleukin6 (IL-6). The trigger for inducing the release of pro-inflammatory cytokines is the activated nuclear factor-κB (NF-κB) pathway via the Toll-like receptor 4 (TLR4) receptor [50]. Despite the extensive description of ENS and inflammation, the specific interconnection is unknown.

### 1.4. Obesity

**Figure 1:**
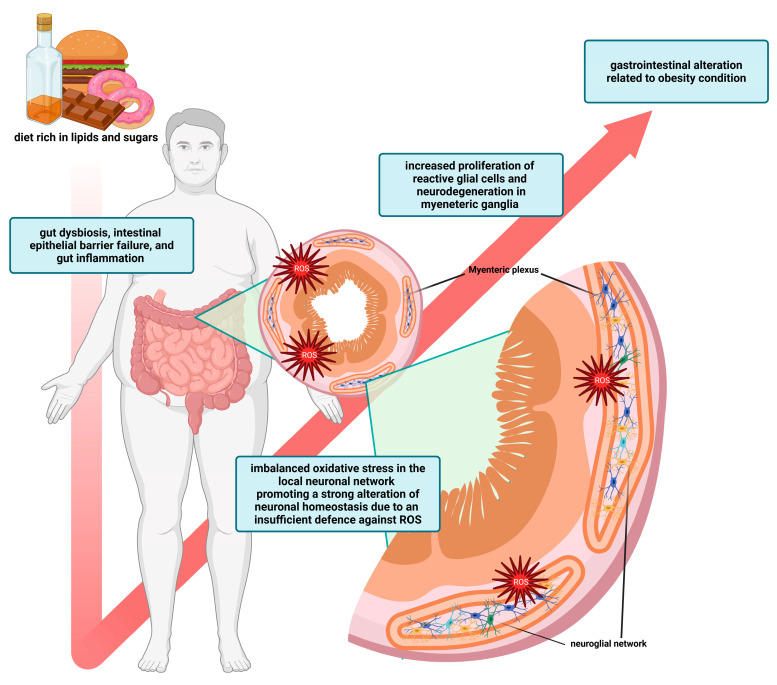
Detrimental effects of obesity-related oxidative stress on intestinal homeostasis. A diet rich in lipids and carbohydrates induces gut dysbiosis and a corresponding intestinal epithelial barrier failure. The local neuronal network along the gut wall undergoes significant alterations due to an imbalanced oxidative status. In this condition, enteric glial cells switch their metabolic rate into a reactive phenotype that increases the local and systemic pro-inflammatory microenvironment. Created in BioRender. Bellitto, V. (2025) https://BioRender.com/2a671ka (15 November 2025).

Recently, it has been documented that ENS is kept apart in the development of obesity-related distress. It also contributes to the complex problem of modulating gut arrangement [7]. A diet rich in lipids and carbohydrates causes oxidative stress and a local pro-inflammatory environment, which leads to insufficient neuronal resistance against ROS and an altered cellular homeostasis of the neuronal network [7,51,52,53] (Figure 1). The prevalence of obesity has risen significantly over the past century, posing a major risk factor for chronic diseases associated with metabolic syndrome, including type 2 diabetes and cardiovascular disease [54,55,56,57]. Epidemiological studies support the link between obesity and alterations in the intestinal tract, but the underlying mechanisms of this association remain poorly understood [58,59]. Rodent models of obesity are commonly employed to investigate the physiological mechanisms behind this pathology. The high-fat diet (HFD) is a well-established model of excess caloric intake and increased fat mass. Other symptoms consistent with those observed in human obese subjects include intestinal problems and dysmotility, likely caused by multiple factors: gut dysbiosis, intestinal epithelial barrier failure, and gut inflammation [53,60,61]. Recently, the ENS has also been considered a target for researchers studying dysmotility, a novel aspect to explore in HFD models, as the ENS appears to be neuroplastic and highly adaptive when exposed to numerous internal and external stimuli. [62,63,64]. Enteric neuroplasticity is the property of the ENS that involves both reparative and maintenance actions to face several gut stimuli to maintain physiological homeostasis [64]. During plastic responses, alterations within the ENS are commonly associated with concomitant structural, chemical, and functional modifications in interconnected neuronal circuits. Studies have reported altered synaptic transmission, changes in neuronal excitability, chemical coding, glial reactivity, and neurodegenerative and neurogenic processes in the neuronal network during pathological conditions like obesity, inflammation, and disease [64]. Frequently, these alterations will affect the gut’s function. As a result, the HFD model may lead to abnormal plasticity in the intestinal wall. Additionally, HFD-induced neurotropic plasticity and dysmotility are proposed to occur before the traditional onset of metabolic syndrome [7,65,66,67,68,69,70,71,72,73]. The altered intestinal motility is a multifactorial problem of both the small intestine and colon; these issues include contractility and relaxation capacity, altered excitability of the smooth muscles, and intestinal dysrhythmia, all of which led to decreased motility [60,65,74]. Abnormal enteric responses have also been associated with an overstimulation of neuronal activity in intrinsic circuitry and projections, which are primarily responsible for regulating movement [2,66]. Different triggers can influence the neural control of the intestine, including the immune and hormonal systems, the gut’s microbiota, and dietary components. As a result, alterations to the ENS are currently paramount to many debates about the causes of HFD-induced dysmotility [2]. These adaptive responses involve neurons in the intestinal wall and other surrounding cells, such as EGCs, immune cells, and intestinal epithelial cells [75]. The enteric neuronal network has an intense metabolic rate and is vulnerable to an unbalanced redox state. As reported in preclinical studies discussed in this review, the neuroglial network and enteric neuronal subpopulations are differentially affected by conditions associated with obesity-related oxidative stress and inflammation, with pronounced morphological modulation and ultrastructural changes [51,52,53,60,61,62,63,64,65,66,67,68,69,70,71,72,73,74,75,76,77,78,79,80,81,82,83,84,85,86,87,88,89]. The main experimental results are summarized in Table 1 [7,60,65,66,67,68,69,72,74,75,76,78,80,81,84,85,89]. 

Studies in animal models reported HFD-induced neuromodulation, especially in the myenteric ganglion (Table 1). Changes in the neuronal composition [67,75], localization and density [67], alterations to the neuromuscular reflex [80], altered ganglion size [75], and alteration of neuronal markers [66,67,72,74,75] are reported in Table 1. The glial network is also affected by obesity-related inflammation, especially in EGCs density [7,67,74,81,84,85]. Neurons that compose the myenteric plexus have direct control over motility and are among the most adversely affected neurons in the HFD model (Table 1). Data indicate that different HFD protocols, regardless of the percentage of fat and sugar in the diet and of the duration of manipulation, can lead to alterations that can promote myenteric neuron loss [68,69,70,72,76,77,78,85] that generate a strong reduction in colonic transit dysmotility and severe gut clinical symptoms [66,67,74,75]. There has been documented axonal swelling, loss of cytoskeletal components, decreased ganglion size, and reduced amounts of nerve cell soma in the myenteric plexus, leading to morphological changes and then apoptotic events [65,72,77]. As already reported, obesity causes imbalanced oxidative stress in the local neuronal network [68,78,80], promoting a strong alteration of neuronal homeostasis due to an insufficient defense against ROS. This damage seems to be independent of the time of HFD. Neuronal electrophysiological properties appear to be disturbed, and neuronal loss is observed in work with short or long manipulation. Moreover, myenteric neurons have a higher response to ACh and serotonin [66,73]; these neurotransmitters are primarily responsible for the peristaltic reflex with a significant impact on the regulation of motility [7,54,67]. Connected with enteric neurons, HFD is also characterized by a reduced excitability of smooth muscle cells, which is possibly related to the altered electrical properties of neurons. Specific subpopulations of enteric neuronal cells are subject to neuromodulation and density loss, like nitrergic, cholinergic, and VIP-ergic neurons. Studies reported an impairment of excitatory and inhibitory neuronal networks with ACh, VIP, and NO, as primary neurotransmitters in this regard [60,65,67,68,69,70,71,72,74,75,79,80,81,84]. Despite reported data suggesting an active implication of enteric neurons in HFD-induced dysmotility, the mechanism by which dissimilar HFD protocol manipulation leads to different effects is unclear. It is plausible that the complexity of each neuronal subpopulation, their sensitivity to other insults, and the amount of oxidative stress and neuroinflammation, may be the reason for the different conclusions observed in these studies. Besides, the molecular mechanisms showing how these insults affect enteric neuronal homeostasis in HFD models have not yet been fully outlined. Also, the involvement of the enteric glial cell network plays an important role in HFD-induced neuromodulation. In fact, ECGs perform a series of functions aimed at maintaining homeostatic functions in the gut, like regulation of the intestinal epithelium’s proliferation, differentiation, and apoptosis by interacting with the innate immune system [82,83]. The immunoreactivity of a glial marker as S100β, would seem an indicator of how HFD affects the enteric glial network. This last one appears with a reduced immunoreactivity in the mucosal layer, representing a decline during HFD-feeding, suggesting that obesity acts as a dysfunctional trigger on the EGCs network [38,39,67]. Conversely, intra-ganglionic EGCs are closely associated with the ENS [41]. This strong interconnection seems to lead to an EGC overactivation [7,67,74]. As a result, the importance of EGCs has been questioned. McClain, J.L. et al. suggest that EGCs are linked to the enteric nervous system’s reflexes that control the movement of the GIT [41]. Similarly, Beraldi et al. reported that inflammation and oxidative stress related to obesity promoted the loss of myenteric glia, accompanied by dysmotility [67]. Also, increased immunoreactivity for S100β in the ganglia has been documented as a common attribute of glial cells during inflammation [45]. However, available research has examined only a few of the markers of inflammation, like IL-1β and IL-6 [7]; thus, it is not known if the EGCs are in response to pathology or just to changes that occur because of adaptation. So, both decreases in glial mass and increases in glial biomarkers are associated with gut diseases. The increase in biomarkers of glial origin, as well as the alterations in cell shape and proliferation, are considered general indicators of glial reactivity. This process is ambiguous, context-dependent, and may indicate glial dysfunction and pro-inflammatory activity [8,81,86,87]. Researchers have documented increased proliferation of myenteric glial networks before the loss of neurons in the ganglia caused by HFD [74]. As a result, EGCs seem to cause neuronal death during colonic inflammation [24]. Because of the intricacy of the neuroglial network, additional studies are necessary to elucidate the onset of these changes and understand how EGCs are involved in the behavioral alterations observed in HFD-fed mice [85,88,89]. As a result, it is possible to deduce that inflammation-induced neuromodulation and the EGC’s role in both processes can play a crucial role in understanding the effects of obesity-related oxidative stress and inflammation using HFD models.

Bona, M.D. et al., [90] recently reported a systematic review that assesses the evidence regarding the association between obesity, metabolic syndrome and alteration of the intestinal barrier permeability in humans. Obese patients display an increase in intestinal epithelial barrier permeability, depletion of goblet cells, reduced mucin glycosylation, and a decreased expression of Zonulin-1, Occludin, and Claudin-1, which form the tight-junction protein between intestinal epithelial cells and that guarantee the correct semi-permeability of the intestinal mucosa [91]. Despite this intriguing aspect, human studies that analyze the correlation between altered intestinal permeability, obesity-related low-grade inflammation, and its effects on the neuronal and glial network and motor dysfunctions are currently lacking. The incidence of obesity and intestinal disease, known as IBD, is increasing worldwide. Several lines of evidence suggest that these conditions may be associated with one another through a shared environmental factor that leads to changes in the intestinal microbiome connected to a low-grade systemic inflammatory state [92]. In accordance with the clinical evidence, the neuro-glial network undergoes plastic changes promoting an exacerbation of critical alterations observed in the digestive tract during inflammatory events. Several studies have reported a strong modulation of neuroglial density in enteric ganglions not restricted to specific neural populations [93,94], promoted by an altered apoptotic event [95,96,97,98], suggesting that neuroinflammation induces enteric neuronal death or altered expressions of enteric neurotransmitters. For instance, low levels of VIP have been observed in the colon of IBD patients [96]. The density of EGCs results in reduced human samples considered, especially for the S100β -positive glial cells [93,98], compromising the correct glial cells/neurons ratio [94]. The reduced density of EGCs can be favorable to an augmented apoptotic rate observed in both enteric plexuses [95]. Other human studies reported EGC abnormalities, especially in the cellular ultrastructure [99]. In support of this view, histological analysis conducted on human intestinal biopsies reports an augmented expression of GFAP and S100β in the inflamed area, and a diminished expression in the non-inflamed tract [97], suggesting that EGCs were connected to the inflammatory processes. The intrinsic pathological mechanisms interplaying the intestinal permeability alteration, immune cell infiltration, pro-inflammatory environment, enteric nervous glial connection, and bowel dysfunctions in IBDs remain to be elucidated.

## 2. Nutrients and Bioactive Compounds as Antioxidant Modulators of ENS in Preclinical and Clinical Studies

**Figure 2:**
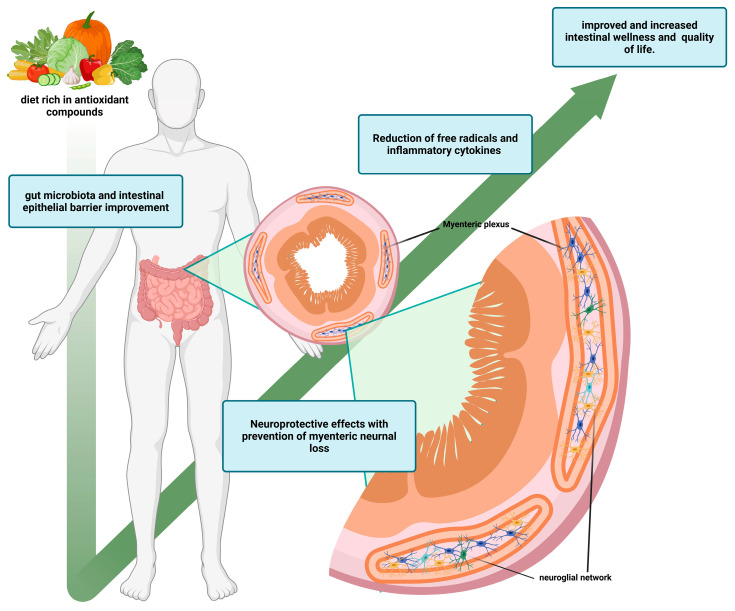
Positive effects of antioxidant compounds on intestinal homeostasis. A diet rich in antioxidant compounds and bioactive molecules promotes intestinal microbiota eubiosis and a related improvement in the intestinal epithelial barrier. Furthermore, it has positive effects on the reduction in free radicals and pro-inflammatory cytokines, resulting in neuroprotective and preventive effects on the neuroglial network, with a positive impact on overall intestinal homeostasis. Created in BioRender. Bellitto, V. (2025) https://BioRender.com/xm9kfvn (accessed on 15 November 2025).

Free radicals are continuously generated in the human organism, which are in part used as triggers to vital responses and in part are neutralized through the antioxidant system composed of enzymatic and non-enzymatic mechanisms [100]. An imbalanced status between oxidant and antioxidant agents, favoring the oxidant molecules, contributes to the development of many diseases. In recent years, the modern lifestyle, overeating junk food, exposure to numerous chemical agents, and physical inactivity play a major role in the induction of the imbalanced oxidative status, also related to obesity related inflammation [101]. Furthermore, those antioxidants that are not only endogenously synthesized but can also be acquired from the diet [102]. In fact, foods rich in vitamins, minerals, and other bioactive compounds can contribute to reinforcing the cellular antioxidant compartment against the excess of free radicals. For this reason, in the past decade, it has emphasized the consumption of food rich in antioxidant compounds as a non-pharmacological approach against all the diseases that originate from an imbalanced oxidant status, like obesity-related oxidative stress and inflammation [103] (Figure 2). Dietary intake may affect the ENS in different metabolic pathways. For instance, by sensing luminal contents, in fact, some amino acids like L-cysteine can participate in ENS transmission, acting as a substrate for NO production [104]. Another example is the influence of glutamate on neuroendocrine mechanisms in the GIT, modulating appetite and intestinal motility and impacting hormone-producing enteroendocrine cells (EECs) [105]. Food that offers physiological benefits has, for years, been popular, largely due to a growing interest in healthy eating. While it is mentioned that some compounds and nutrients have proven antioxidant effects in vitro, such as bioactive peptides, phenolic compounds, isoflavones, carotenoids, tocopherols, phytosterols, and their effect on disease prevention, especially concerning the modulation of oxidative stress markers, and their relationship with the supplemented dose is not well established [106]. Therefore, to better understand the possible positive effects of antioxidant compounds on individual health, and their bioavailability and bioactivity, there is a need to investigate the effects of different bioactive molecules, through preclinical and clinical studies, including in vivo experiments using animal models as well as trials involving humans. This review presents and discusses the neuroprotective properties of the most common compounds that interfere with the oxidative status of the ENS in relation to preclinical studies (Table 2) using experimental animal models [107,108,109,110,111,112,113,114,115,116,117,118,119,120,121,122,123,124,125,126,127,128,129,130,131]. Essentially, it results in a reduction in protein nitration inside the enteric plexus [107,108,109,110], prevention from neuronal loss [109], an increment of myenteric neuronal density [111], reduction in morphological changes [112,113], and a decrement of oxidative damage on neuronal sub-population [114,115,116,117,118,119]. However, studies conducted on the effects of the intake of exogenous natural antioxidant compounds on the endogenous antioxidant defense system of human metabolism are still unclear, and even less clear are the possible beneficial effects of antioxidant supplementation on the ENS. In fact, in the recent scientific literature, there are interesting clinical studies [132,133,134,135,136,137] conducted on patients with intestinal disorders, especially Ulcerative Colitis (UC) and Crohn’s Disease (CD), in which were demonstrated the antioxidants capacity of many natural bioactive compounds with a reduction in oxidative stress and inflammatory conditions but there is a lack of evidence in terms of interaction of these antioxidants’ compounds and the ENS (Table 3).

### 2.1. L-Glutathione

L-glutathione is an essential non-enzymatic antioxidant in mammalian cells that acts as a direct antioxidant, protecting cells from free radicals and pro-oxidants, and as a cofactor for antioxidant and detoxifying enzymes such as glutathione peroxidase, glutathione S-transferase, and glyoxalase. In Table 2, a reduction in total antioxidant capacity and catalase (CAT) activity was reported in the intestines of a rodent model of diabetes. The authors therefore speculated that the improvement in local antioxidant defence induced by L-glutathione increased glutathione levels to the point where it was not necessary to enhance other antioxidant mechanisms, such as CAT. They also reported a decrease in the concentrations of hydroperoxides, lipid peroxides, and nitric oxide, confirming a reduction in oxidative stress. This improvement was reflected by a decrease in the number of myenteric neurons labelling nitro-tyrosine [107]. Glutathione is endogenously present in a variety of plant-based foods, including asparagus, avocado, and cucumber. Cruciferous vegetables rich in sulphur, such as broccoli, kale, and cauliflower, also represent significant dietary sources of this compound. Glutathione is classified as “generally recognized as safe” for use in food products; however, supplementation may elicit adverse reactions in susceptible individuals. Oral administration at doses of up to 500 mg per day for periods of up to two months is considered possibly safe. Reported adverse effects are generally mild and include headache, abdominal discomfort, bloating, nausea, and diarrhoea [138].

### 2.2. Resveratrol

Resveratrol (RSV) is a natural compound (3,5,4’-trihydroxy-trans-stilbene) found in grape skins, red wine, blueberries, and peanuts. Its biological properties—mainly antioxidant, anti-inflammatory, anticancer, estrogenic, vasorelaxant, and cardioprotective activities—are of great relevance in medicine and pharmacy [108]. Several preclinical and clinical studies have evaluated the antioxidant potential of RSV supplementation in mitigating oxidative stress and promoting remission in various human pathologies characterized by high oxidative imbalance. Locally, RSV can counteract the overactivity of oxidative enzymes, thereby reducing oxidative and nitrosative stress, enhancing the activity of antioxidant enzymes such as superoxide dismutase (SOD) and glutathione-S-transferase (GST), lowering lipid peroxidation, and decreasing blood levels of MDA and TNF-α, thus improving the total antioxidant status [109,133]. In animal models of intestinal ischemia, RSV administration—either before or after surgery—was shown to reduce oxidative stress and free radical formation, decrease protein carbonylation and lipid peroxidation, restore glutathione balance, and increase the activity of antioxidant enzymes such as GPx, SOD, GSH, and glucose-6-phosphate dehydrogenase [110,111,112,113]. Positive effects of RSV supplementation on the ENS were also observed, with overall neuroprotection reported by all studies summarized in Table 3. Myenteric neuronal density increased, while nitrated protein accumulation decreased, suggesting reduced neurotoxicity from oxidative stress. The nitrergic neuronal subpopulation, which is particularly sensitive, appeared less affected [111,112,113]. Moreover, RSV supplementation reduced glial reactivity and hyperproliferation of EGCs [111,112,113]. Similar effects were found in animals treated with a chemotherapeutic drug, where RSV appeared to reverse nitrated protein translocation in myenteric neurons, reducing neurodegeneration and apoptosis [110]. Despite variability in dosage, routes of administration, potential gastrointestinal adverse effects, and interactions with concomitant therapies, RSV treatment has not been consistently associated with clinically significant adverse events. Overall, its substantial therapeutic potential in degenerative disorders, inflammatory diseases, and a wide range of chronic conditions warrants considerable attention. Nevertheless, current evidence remains insufficient to draw definitive conclusions. Well-designed, large-scale randomized controlled trials are therefore needed to define optimal dosage and treatment duration, comprehensively assess safety and pharmacological interactions, and evaluate both short- and long-term outcomes of RSV across different age groups, with particular emphasis on the elderly population [139].

### 2.3. Quercetin

Quercetin (QE), 2-(3,4-dihydroxyphenyl)-3,5,7-trihydroxychromen-4-one a 3,3′,4′,5,7-pentahydroxyflavone, is one of the flavonols commonly found in fruits and vegetables such as apples, onions, and berries, and belongs to the large class of flavonoid polyphenols. Previous studies have investigated the potential antioxidant effects of QE supplementation on ENS cells under pathological conditions [114]. In an animal model of diabetic rats, QE modulates the ENS by decreasing protein carbonylation, lipid peroxidation, and ROS overproduction, while increasing total antioxidant capacity and the activity of antioxidant enzymes such as GPx and SOD in the small intestine [115,116]. The counteraction of oxidative status by QE supplementation counteracts oxidative stress, promoting neuroprotection, especially in nNOS-positive [116] and VIP-positive neurons [117]. From a neuroimmune perspective, Vieira-Frez et al. [118] also reported that quercetin was able to modulate the population of resident M2-like macrophages responsible for releasing anti-inflammatory and reparative cytokines. QE has been shown to exert pronounced anti-inflammatory effects, primarily through the inhibition of pro-inflammatory cytokine production, downregulation of cyclooxygenase and lipoxygenase expression, and stabilization of mast cell integrity. By modulating oxidative stress and inflammatory pathways, QE has demonstrated potential therapeutic efficacy across a wide range of pathological conditions. Although antioxidant-based strategies have been proposed to mitigate excessive oxidative and inflammatory damage, limitations related to cost, accessibility, and adverse effects persist. In this context, natural compounds—particularly plant-derived products—represent widely available, cost-effective, and generally well-tolerated alternatives [140].

### 2.4. Inulin

Inulin, a soluble dietary fiber, is widely distributed in more than 36,000 plant species, where it serves as a polysaccharide reserve. The primary sources of inulin include chicory, onion, garlic, and barley. It is well established that inulin, as a prebiotic, exerts significant effects on the regulation of the intestinal microbiota by stimulating the growth of beneficial bacteria. In addition, inulin exhibits various health benefits, including the regulation of lipid metabolism, promotion of weight loss, reduction in blood glucose levels, inhibition of inflammatory factor expression, and a decreased risk of colon cancer [119]. The prebiotic potential of inulin is further demonstrated by its ability to increase the concentration of short-chain fatty acids (SCFAs) in the GIT lumen.

Targeting the interplay between dietary toxicants, nutrition, and gut immunity through interventions such as inulin or other fermentable fibers may help attenuate the metabolic disruptions induced by these compounds. Elucidating the causal mechanisms within the microbe–host axis that drive toxicant-induced dysmetabolism, therefore, remains a key research priority. Increasing evidence indicates that widely encountered toxicants—including persistent organic pollutants, pesticides, fungicides, and food additives such as emulsifiers and artificial sweeteners—can alter gut microbiota composition and promote the development of metabolic diseases [141]. However, robust evidence regarding the effects of isolated dietary fibers on physiological outcomes, such as laxation, colonic transit time, and postprandial glycemic and insulinemic responses, remains limited, particularly in pediatric populations [142].

Beyond their roles in ROS mitigation and modulation of antioxidant enzyme activity, SCFAs also stimulate enteric neuronal signalling, making inulin supplementation a potential therapeutic approach for modulating ENS responses [120]. Studies on inulin supplementation in HFD rats have reported an improvement in gut microbiota composition and a reduction in pro-inflammatory cytokines such as IL-6. Focusing on ENS modulation, a decrease in gliosis and a reduction in GFAP-positive EGCs have also been observed [121]. Although markers of oxidative stress have not been investigated, the modulatory effects of inulin on ENS function may depend on other factors, such as the status of the intestinal microbiota. Since no studies have yet explored prebiotics as therapeutic interventions for diseases associated with ENS neurodegeneration or dysfunction, this field warrants further investigation.

### 2.5. Ascorbic Acid

Vitamin C [ascorbic acid (AA)] is a vitamin with strong physiological antioxidant activity, protecting organisms by participating in redox balance and directly scavenging ROS. In general, AA is a well-recognized antioxidant capable of neutralizing free radicals, particularly under conditions associated with aging and chronic diseases such as cardiovascular disorders and certain cancers. Through its antioxidant activity, AA contributes to the protection of DNA, proteins, and lipids from oxidative damage. Observational studies suggest that higher AA intake, especially from dietary sources, may be associated with a reduced risk of cardiovascular disease, potentially due to its protective effects on vascular function and its ability to reduce oxidative stress. In contrast, high doses of vitamin C—most commonly from supplementation and exceeding the tolerable upper intake level of approximately 2000 mg/day—may induce adverse gastrointestinal effects, including nausea, diarrhea, and abdominal cramps, due to its water solubility and osmotic properties. Moreover, excessive AA intake may increase urinary oxalate levels, thereby contributing to kidney stone formation in susceptible individuals [143]. Despite the promising potential of vitamin C in protecting the ENS, and AA being full of potential, the effects of this supplementation on oxidative stress remain poorly investigated. However, De Freitas et al. [122] and Zanoni et al. [123] examined the effects of this micronutrient on myenteric neurons in an animal model of diabetes affecting the small intestine. Both studies observed that AA had exerted a protective effect on the intestinal mucosa and was able to restore the morphology and density of the enteric neuronal population, with an increase in VIP-ergic neurons. Data are also currently lacking regarding the impact of these responses on gut motility, muscular contractions, and the broader panorama of functional outcomes associated with AA supplementation. Furthermore, de Freitas et al. [124] investigated the modulatory effects of AA on the ENS of healthy and aged rats to evaluate neurodegeneration induced by age-related oxidative stress. No alterations were observed in the myenteric neuronal population of the small intestine, and therefore, a neuroprotective activity was ascribed to AA. The lack of direct evaluations of oxidative stress markers or antioxidant capacity in the intestinal tract highlights the need for further studies to confirm and explore this hypothesis.

### 2.6. L-Glutamine

Glutamine is a non-essential amino acid present in foods and animal-derived by-products. It participates in multiple cellular physiological pathways, particularly those related to immune function. Glutamine is generally considered safe; however, mild adverse effects may occur, which typically do not require medical intervention and often resolve with continued use. Under conditions of intense stress or catabolism, glutamine becomes a conditionally essential amino acid. In the gut, it serves as an energy substrate that supports enterocyte proliferation and differentiation [125]. Supplementation with different concentrations (1% or 2%) of L-glutamine in diabetic Wistar rats has generally shown neuroprotective effects [126,127]. The authors suggest that supplementation may exert these effects by restoring non-enzymatic cellular antioxidants, such as GSH, which directly react with ROS [128]. Neuroprotection was evidenced by the preservation of neuronal density in the myenteric plexus and a reduction in neuronal death that is otherwise prominent in diabetic groups. Furthermore, it was reported that 2% L-glutamine supplementation counteracted glial reactivity, reducing glial hypertrophy and normalizing ganglion density [127]. Since L-glutamine can restore GSH levels, the enhancement of cellular antioxidant defenses may explain how this amino acid modulates glial behavior [129]. However, further studies are needed to clarify the effects of glutamine on the neuroplasticity of specific neuronal subpopulations within the myenteric plexus and on general gut physiological responses.

### 2.7. Ginger

Ginger [*Zingiber officinale* (GE)] is a flowering plant whose rhizome—commonly known as ginger root—is widely used as both a spice and a traditional remedy in folk medicine. The GE, in fact, has potent antioxidant activity, as demonstrated in preclinical and clinical studies [130,131,132,137].

Nevertheless, ginger has a long history of use as both a food and a medicinal product and is classified by the U.S. Food and Drug Administration as “generally recognized as safe” when used as a food flavoring, including during lactation. When consumed for medicinal purposes, ginger is generally well tolerated in adults; however, mild adverse effects—such as unpleasant taste, heartburn, abdominal discomfort, weight gain, headache, dry mouth, and nausea—have occasionally been reported. Additionally, ginger may increase bleeding risk when co-administered with warfarin [144,145].

Preclinical evidence suggests that supplementation with GE extract prevented dopaminergic enteric neuronal loss in an animal model of Parkinson’s disease [130,131]. The enteric neuroprotection observed in this research can be associated with the antioxidant activity of GE, including increased SOD activity and decreased lipid peroxidation and protein carbonylation [132]. Although EGCs were not investigated in this study, supplementation was reported to improve the intestinal epithelial barrier, with an increase in tight junction proteins and a decrease in inflammatory cytokine levels. These effects could indicate an improved glial response induced by ginger supplementation. Preservation of a physiological intestinal epithelial barrier could indirectly counteract gut oxidative stress by reducing inflammation and ROS generation [131].

### 2.8. Curcumin

Curcumin, a natural compound extracted from the rhizomes of *Curcuma longa*, has a long history of use in traditional medicine, particularly across Asia. In recent years, it has gained significant scientific attention for its diverse biological activities, including antioxidant, anti-inflammatory, and anti-carcinogenic effects. These properties have led to ongoing research into its potential role in the prevention and treatment of various inflammation-related diseases [146]. Curcumin attenuates the intensity of the disease process, leading to a reduction in the DAI score and an improvement in disease remission [133]. These beneficial effects are thought to arise from multiple molecular mechanisms. One proposed mechanism involves its ability to inhibit the activation of transcription factors such as NF-κB and STAT3, which play key roles in regulating the expression of pro-inflammatory cytokines [147]. Overall, curcumin has demonstrated potential benefits in obesity management by attenuating inflammation, inhibiting adipose tissue expansion, and improving metabolic markers. Several studies have reported reductions in body weight, body mass index, and waist circumference; however, results are not consistent across all trials. Although generally well tolerated, high doses of curcumin may cause mild gastrointestinal side effects, such as diarrhea. Overall, curcumin appears to be a relatively safe, natural supplement that may serve as an adjunct or alternative to certain pharmacological treatments [148].

### 2.9. Other Bioactive Compounds

#### 2.9.1. Genistein

Genistein is an isoflavone originally isolated from the flowering plant *Genista tinctoria* L., which is commonly found in species belonging to the Fabaceae family. In mammals, genistein exhibits estrogen-like activity due to its structural similarity to estrogen. Preclinical studies have shown that genistein possesses multiple biological properties, including antioxidant and anti-inflammatory activities, and can influence hormonal activity, glucose regulation, and lipid metabolism [149]. Isoflavones were generally well tolerated across all the studies reviewed. Reported adverse effects mainly involved the gastrointestinal system and may include hormonal implications due to their estrogen-like properties [150]. As shown in Table 3, genistein treatment significantly modulated serum levels of adiponectin, leptin, and TNF-α in patients, confirming its anti-inflammatory and metabolic effects [134].

#### 2.9.2. Silymarin

Silymarin, derived from the seeds of the milk thistle plant, has a long history of use in managing various health conditions. It functions as an antioxidant by neutralizing free radicals and modulating enzymes involved in cell damage and fibrosis [151]. It is best known for its hepatoprotective effects. In addition, studies have reported other therapeutic properties, including anticancer, neuroprotective effects and antidiabetic activities, making its safety profile particularly important. No major toxicity has been observed in animal studies. Silymarin is considered safe in humans at therapeutic doses and remains well tolerated even at high doses of 700 mg administered three times daily for up to 24 weeks. Reported adverse effects were limited to mild gastrointestinal discomfort. Additionally, one clinical trial indicated that silymarin is safe during pregnancy, with no associated fetal anomalies observed [152]. These antioxidant effects were observed in clinical studies on pediatric Crohn’s disease (CD), where silymarin treatment was associated with improved oxidative stress parameters and a significant reduction in pro-inflammatory cytokines. Moreover, silymarin also increased antioxidant markers such as SOD and TAC [135].

#### 2.9.3. Berberine

Berberine is a naturally occurring alkaloid found in various medicinal plants. Due to its unique chemical structure, it exhibits a wide range of biological activities, including antioxidant, anti-inflammatory, cholesterol-lowering, antidiabetic, anti-obesity, and antimicrobial effects.

Berberine exhibits neuroprotective effects in different neurodegenerative and neuropsychiatric disorders. Despite its low oral bioavailability, Berberine remains a promising therapeutic candidate for multiple conditions, potentially through modulation of the gut microbiota. Although evidence related to the aging process in humans is limited, preliminary studies have demonstrated beneficial effects across various experimental models [153]. Furthermore, recent studies have explored its potential in combating different types of cancer, suggesting possible roles in the prevention and treatment of malignancies such as breast, lung, gastrointestinal, liver, and colorectal cancers [154]. In a double-blind clinical trial involving biopsy-proven ulcerative colitis patients, treatment with 900 mg/day of berberine for 3 months significantly reduced local inflammation and decreased pro-inflammatory cytokine levels [136]. In general, orally administered berberine is considered well-tolerated; however, it is associated with certain risks and adverse effects.

## 3. Future Prospective

Although findings from existing studies are often controversial or inconclusive, emerging evidence from preclinical research and early clinical trials suggests that targeted dietary interventions may help mitigate disease-related symptoms and treatment-associated toxicities. Potential mechanisms underlying these effects include modulation of inflammation and oxidative stress, preservation of muscle mass, improvement of cardiovascular health, and regulation of the gut microbiota. The molecular mechanisms underlying intestinal cell dysfunction in obesity are still poorly characterized; however, an emerging and promising approach is the use of intestinal organoids to investigate lipotoxicity in intestinal cells [155]. Ongoing studies are expected to further clarify whether specific dietary strategies, particularly those targeting the gut microbiota, can effectively improve treatment outcomes. Future research should also investigate the synergistic effects of combined nutritional interventions and support the development of evidence-based dietary guidelines for the management of gastrointestinal disorders related to obesity.

In this context, it is evident that nutrients and bioactive compounds can positively influence intestinal homeostasis and overall health. Nutritional epigenetics is a branch of epigenetics that examines how dietary lifestyle and specific nutrients affect protective epigenetic modifications throughout life. Bioactive compounds can act directly by inhibiting epigenetic enzymes such as DNA methyltransferases, histone deacetylases, and histone acetyltransferases, thereby altering DNA methylation status, histone modifications, and chromatin remodeling, or by changing the availability of substrates required for those enzymatic reactions. These effects can alter the expression of key genes and impact overall health and longevity [156]. For example, RSV, known for its strong antioxidant, anti-inflammatory, anticancer, and cardioprotective properties, acts epigenetically by removing acetyl groups from histones, thereby improving health [157].

However, nutritional epigenetics is a relatively recent subfield, so current knowledge of the possible effects of bioactive nutrients on epigenetics and their associations with health homeostasis and diseases is limited. Considering the evidence collected in this literature review and the recent study on nutritional epigenetics, the potential clinical impact of the natural antioxidant compounds discussed could pave the way for personalized nutritional interventions that promote health by reducing the oxidative stress and inflammation associated with diseases, such as obesity [158].

## 4. Conclusions

**Figure 3:**
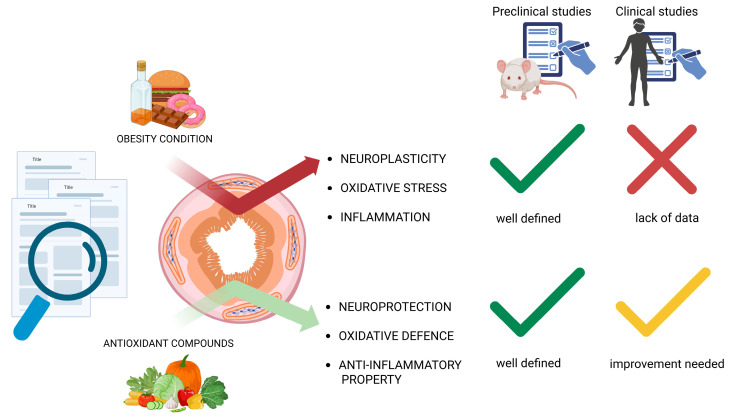
Reviewing the scientific literature relative to the effects of obesity-related oxidative stress on the ENS and any potential improvements promoted by antioxidant compounds, it emerges that numerous interesting studies have been carried out at a preclinical level using animal models, while as regards clinical studies, a lack of evidence is evident, especially considering the analyses of the ENS on specimens from obese patients with intestinal problems. Created in BioRender. Bellitto, V. (2025) https://BioRender.com/tttz2ye (accessed on 15 November 2025).

Myenteric plexus neuroplasticity is an important field that deserves further exploration to better identify obesity-related dysmotility. In this emerging research area, the present review collects scientific evidence from numerous preclinical studies using different animal models, highlighting the detrimental effects of HFD and obesity on the sensitive and complex interconnections between enteric neurons and EGCs. This literature review clearly demonstrates the lack of significant data from human studies regarding the pathological effects of obesity on the ENS. Indeed, all the available clinical data are derived from biopsies of patients with intestinal disorders such as IBD and are therefore not strictly related to obesity. Significant progress has been made in understanding the antioxidant effects of nutrients and bioactive compounds on the ENS, but several mechanistic aspects remain to be clarified, particularly through clinical studies. The detailed signaling pathways between myenteric neurons, EGCs, and neurotrophic mediators through which these molecules improve the oxidative balance within the myenteric plexus have not yet been fully elucidated. Moreover, little is known about the interactions between neural elements and other intestinal cell types, including epithelial, immune, and enteroendocrine cells, which might represent an additional level of antioxidant response worth investigating. Experimental evidence discussed in this review indicates that nutrients and bioactive compounds can attenuate oxidative stress in the ENS by enhancing endogenous antioxidant defenses and supporting the activity of neuroprotective factors, specific enzymes, and neurotrophins. However, most of this evidence comes from preclinical studies—animal or in vitro—and translational data in humans remain limited, particularly regarding the optimal and safe levels of supplementation. Nevertheless, nutritional interventions appear to offer safety advantages due to their lower toxicity compared to pharmacological strategies. Further clinical investigations are therefore needed to clarify how these compounds modulate oxidative stress and to evaluate their therapeutic potential in disorders that compromise the “second brain” (Figure 3).

## Figures and Tables

**Table 1:** Obesity-related oxidative stress and inflammation insults on ENS, in vivo evidence.

Animal Model/Age	Manipulation Time	Diet	Gut Insults	ENS Modulation	EGCs Modulation	Ref.
C57BL/6J mice 4 weeks old	24 weeks	HFD with 45% of fat	↑ IL-1β ↑ IL-6 ↑ MDA ↓ Occludin ↑ Local inflammation	↓ Myenteric neurons	↑ Overactivation of EGCs ↑ Density of EGCs in myenteric ganglia	[7]
C57BL/6 mice 8 weeks old	13 weeks	HFD with 60% of fat	↓ Transit ↓ Motility	↓ Nitrergic myenteric neurons	Not analysed	[60]
C57BL/6 mice 8 weeks old	8 weeks	HFD with 72% of fat	↓ Intestinal propulsive motility ↑ Dysbiosis	↓ Inhibitory neuromuscular transmission ↓ Nitrergic myenteric neurons Axonal swelling ↓ Neuronal cytoskeleton	Not analysed	[65]
C57BL/6 mice 4 weeks old	12 weeks	DIO with 61% of fat	↑ Colonic transit -Altered GUT function independent by inflammation	↑ Up-regulation and enhanced signalling of ACh and serotonin No impairing of mucosal integrity No immune cells infiltration	Not analysed	[66]
Swisse mice 7 weeks old	17 weeks	HFD with 59% of fat	↓ Colonic motility -No chance in pro-inflammatory cytokines	↓ Myenteric neurons ↓ Myosin V-neurons ↓ VIP-ergic neurons ↓ Nitrergic neurons	↓ Glial cells density	[67]
Germ free Swisse mice 7 weeks old	8 weeks	DIO with 60% of fat	↑ OS ↑ TNF-α in myenteric ganglia	↓ Total number of myenteric neurons ↓ Density of nitrergic neurons ↓ Nitrergic myenteric neurons	Not analysed	[68]
C57BL/6 mice 3 weeks old	9–12 weeks	HFD with 60% of fat	-Motility disorders -Constipation -GUT microbiota dysbiosis	↓ Nitrergic myenteric neurons ↓ In vitro neurodegeneration ↑ Apoptosis in cultured myenteric neurons	Not analysed	[69]
C57BL/6 mice 7 weeks old	8 weeks	HFD with 72% of fat	↑ Dysmotility ↑ GI symptoms	↓ Myenteric neurons Axonal swelling ↓ Neurofilaments ↓ VIP-ergic neurons ↓ Nitrergic neurons No change in ChAT neurons No change in Substance P levels	Not analysed	[72]
Sprague Dawley rats 6 weeks old	6–12 weeks	HFD with 60% of fat	↑ Peripheral inflammation	↓ Myenteric neurons ↓ Nitrergic myenteric neurons ↓ PGP-Immunoreactive neurons No change in ganglion size	↑ Glial density ↑ Glial proliferation	[74]
C57BL/6 mice 7 weeks old	8 weeks	HFD with 72% of fat	-Motility disorders	Compromised nerve health ↓ Ganglion size ↓ Cell soma areas ↓ VIP-ergic neurons ↓ ChAT neurons ↓ Nitrergic neurons density ↑ Nitrergic’s cell bodies in ganglia ↑ Nitrergic and cholinergic neurons apoptosis	Not analysed	[75]
Swisse mice 7 weeks old	8–17 weeks	HFD with 35% of fat	↓ Motility ↑ Visceral fat	↓ Myenteric neuronal density ↓ VIP-ergic neurons ↓ Nitrergic neurons ↓ Neuronal soma areas	Not analysed	[76]
C57BL/6 mice 4 weeks old	24 weeks	HFD with 45% of fat	↑ OS	↓ Myenteric neurons ↑ Neuronal shrinkage	No changes in glial survival	[78]
C57BL/6 mice 16–17 weeks old	22 weeks	HFD with 60% of fat	↑ OS in myenteric plexus ↓ Motility ↓ Circular muscle inhibitory function	↓ Nitrergic neurons ↓ NO levels	Not analysed	[80]
Zucker rats 8 weeks old	20 weeks	Rat chow diet	Modification of glucidic profile of intestinal secretory product	↑ Progressive neuronal degeneration ↓ nNOS reactive fibers	↑ Reactivity of GFAP-positive EGCs network	[81]
Male Wistar rats 7 weeks old	17 weeks	HFD with 45% of fat	Dysmotility and gastrointestinal alterations	↑ Neurodegeneration of myenteric neurons Alterations of cholinergic and nitrergic neuronal subpopulation	Alteration of EGCs activity due to lipotoxicity	[84]
3 animal models of obesity: Zucker rats and Wistar rats 7 weeks old	-20/17/12 weeks	Rat chow diet, HFD with 45% of fat, cafeteria diet	Intestinal dysbiosis and increment of permeability	↑ Neurodegeneration ↓ Neuronal markers expression Changes in cholinergic and nitrergic networks	Altered immunoreactivity of GFAP-positive EGCs	[85]
C57BL/6 mice 6–8 weeks old	12 weeks	HFD with 60% of fat	↓ Motility	↓ Myenteric neurons ↓ Nitrergic neurons	↑ GFAP-positive EGCs network EGCs overactivation	[89]

Abbreviations: ↑: increment; ↓: decrement; ENS: Enteric Nervous System; ECGs: enteric glial cells; HFD: High-Fat Diet; IL-1β: Interleukin-1-beta; IL-6: Interleukin-6; MDA: malondialdehyde; VIP: Vasoactive Intestinal peptide; ChAT: Choline acetyltransferase; DIO: Diet Induced Obesity; ACh: Acetylcholine; OS: Oxidative Stress; TNF-α: Tumor Necrosis Factor-alpha; PGP-n: Protein Gene Product neurons; NO: Nitric Oxide; GFAP: Glial Fibrillary acidic Protein.

**Table 2:** Antioxidant Activity of Natural and Bioactive Compounds on The Enteric Nervous System in preclinical Studies.

Animal Model/Age/Number	Type of Supplementation and Time of Manipulation	Dose of Bioactive Compound Administered	General Gut Benefits	ENS Modulation	EGCs Modulation	Ref.
Wistar rats DM model, 90 days old *n* = 24	Supplemented normoglycemic diet/120 days	1% L-glutathione	↑ TAC ↑ Catalase activity ↓ Oxidative stress	↓ Protein nitration in myenteric neurons	Not analysed	[107]
Wistar rats DM model, 88 days old *n* = 24	Supplemented rat chow diet/120 days	10 mg/kg of RSV	↓ Oxidative and nitrosative stress	Prevent neuronal loss ↓ Neuronal hypertrophy ↓ nNos positive neurons	↓ ECGs hyperactivation	[109]
Male BALB/c II model after chemotherapeutic administration, mice 5–8 weeks old *n* = 20	3 weeks	Injection of 500 µg/kg daily of RSV	↓ Damage to intestinal mucosa, crypts and muscle layer ↑ Contractility ↓ Constipation	↓ Myenteric neurons with the translocation into nuclei of nitrated proteins ↓ Neurotoxicity	Not analysed	[110]
Wistar rats, II model *n* = 42	Daily gavage for 4 days before the surgery and 7 days later	10 mg/kg of RSV	↓ Oxidative stress ↑ Neuroprotective effects	↑ Myenteric neuronal density ↓ Morphometric changes in myenteric nitrergic subpopulation	↓ Hyperproliferation of EGCs	[111]
Wistar rats, II model *n* = 25	Orally administrated daily before surgery	10 mg/kg of RSV	↓ Lipoperoxidation and carbonylation of proteins ↑ Reduced/oxidized glutathione ratio	↓ Morphological changes in Myenteric neurons ↓ Nitrergic neurons alterations	Not analysed	[112]
Male Wistar rats *n* = 54	standard rodent chow and daily gavage for 5 days before surgery and continued for 7 days after with	7 mg/kg RSV (in free or nano encapsulated form)	↓ Free radicals ↑ Synthesis of antioxidant enzymes ↑ SOD and GSH activity ↓ GSH activity ↑ Gastrointestinal transit	↓ Myenteric neuronal loss ↑ Nitrergic neuronal profile	↓ Glial cell proliferation, ↓ Reactive gliosis	[113]
Wistar rats, DM model 90 days old *n* = 36	Rat chow diet with daily gavage/60 days	QE at 10/100 mg/kg	↓ Carbonylic proteins	Not prevent the overall neuronal loss in general and the cholinergic subpopulation ↓ Oxidative damage of nitrergic population	Not analysed	[115]
Male Wistar rats DM model, 90-day-old *n* = 32	Supplemented normoglycemic diet/120 days	40 mg daily of QE	↓ ROS, ↓ Lipid peroxidation	protective effects on ICC-MY ↑ Nitrergic neurons expression	Not analysed	[116]
Male Wistar rats, DM model 90-day-old *n* = 32	Supplemented normoglycemic diet/120 days	40 mg/day/rat of QE	↑ Bioavailability of NO	↓ Cell body area (swelling) of nitrergic and VIP-ergic neurons indirectly induces neuroplasticity in VIP-ergic neurons.	Not analysed	[117]
Male Wistar rats DM model, 90-day-old *n* = 36	Standard normoglycemic rat chow diet and gavage every day from the 4 days after DM induction	QE stabilized by microencapsulation at 10/100 mg kg^−1^	↓ Oxidative stress ↓ Infiltration of M2-like macrophages	Protective effects on ICC-MY and on nNOS positive neurons	Not analysed	[118]
Male Wistar rats, DM model 6 weeks old *n* = 30	Supplemented HFD (58% fat)/8 weeks	5% Inulin alone or in combination with a probiotic strain	↑ Gut microbiota composition ↓ Levels of inflammatory cytokines	Not analysed	↓ GFAP-positive EGCs ↓ Reactive gliosis	[121]
Wistar rats, DM model 90-day-old *n* = 30	Supplemented normoglycemic diet/120 days	1 g/L daily of AA	No alteration in cellular proliferation of the mucosa	↑ Neuronal density ↑ Neuronal body area	Not analysed	[122]
Wistar rats, 90-day-old *n* = 15	Supplemented standard rat chow diet/120 days	1 g/L daily of AA	No alteration in ileum mucosa	↑ Cellular profile of VIP-ergic neurons	Not analysed	[123]
Male Wistar rats, Aging model 428-day-old *n* = 25	Supplemented standard rat chow diet/120 days	1 mg/mL daily of AA	↓ Free radicals ↑ Neuroprotection	No alteration in cellular profile of VIP-ergic neurons	Not analysed	[124]
Male Wistar rats, DM model 90-day-old *n* = 25	Supplemented normoglycemic diet/120 days	1% daily of L-glutamine	↑ Neuroprotection	Preservation of neuronal density and cell body area	Not analysed	[126]
Wistar rats, DM model 90-day-old *n* = 20	Supplemented normoglycemic diet/120 days	2% daily of L-glutamine	↑ Neuroprotection	Preservation of neuronal death induced by DM	↓ Glial cells size ↓ Ganglion glial density ↑ Gliatrophic effect	[127]
C57BL/6 mice PD model, 7 weeks old *n* = 36	daily gavage/15 days	30/100/300 mg/kg/day of GE	↓ Intestinal tight junctions’ depletion ↑Iintestinal integrity ↓ Inflammatory markers	↑ Neurons survival pathway ↑ Enteric dopaminergic neurons	Not analysed	[130]
C57BL/6 mice PD model, 7 weeks old *n* = 32	Oral administration with daily gavage/22 days	300 mg/kg/day of GE	↓ Inflammatory markers ↓ Intestinal barrier disruption	↓ Degeneration of dopaminergic neurons ↓ Neuroinflammation ↓ Motor dysfunction	Not analysed	[131]

Abbreviations: ↑: increment; ↓: decrement; ENS: Enteric Nervous System; ECGs: enteric glial cells; DM: Diabetes Mellitus; RSV: resveratrol; SOD: Superoxide Dismutase; GSH: Reduced glutathione; QE: quercetin; TAC: total antioxidant capacity; II: Intestinal Ischemia; ROS: reactive oxygen species; ICC-MY: Myenteric interstitial cells of Cajal; GE: Ginger; PD: Parkinson’s disease; HFD: High-Fat Diet; GFAP: Glial Fibrillary acid Protein; AA: Ascorbic acid; VIP: Vasoactive Intestinal peptide; LA: Alpha-lipoic acid; NO: Nitric Oxide; IL-1β: Interleukin-1-beta; IL-6: Interleukin-6; MDA: malondialdehyde; ChAT: Choline acetyltransferase; DIO: Diet-Induced Obesity; ACh: Acetylcholine; TNF-α: Tumor Necrosis Factor-alpha.

**Table 3:** Natural and Bioactive Compound Antioxidant Activity Evaluated in Clinical Studies.

Number of Patients and Duration of Treatments	Antioxidant Natural Compounds	Gut Pathology/ Disturbance	Protective Effects	Antioxidant and Anti-Inflammatory Effects	ENS and EGCs Modulation	Ref.
56 Patients for 6 weeks	RSV	Mild-to-moderate UC activity	Amelioration of clinical condition of disease with the reduction in DAI score	↑ SOD ↑ Total Antioxidant Status ↓ MDA level in blood ↓ TNF-α	Not reported	[133]
45 patients for 8 weeks.	Curcumin	Mild-to-moderate UC activity	Reduction in DAI and improvement of disease remission	↓ Pro-inflammatory cytokines	Not reported	[133]
87 obese postmenopausal women along 6 months	genistein	Obesity (no indication of gastrointestinal problems)	Significant modulation of mean serum levels of adiponectin and leptin.	↓ TNF-α	Not reported	[134]
15 paediatric CD patients for 10 weeks of administration.	Silymarin	CD	Positively influenced the parameters of OS	↑ SOD ↑ TAC ↓ Pro-inflammatory cytokines	Not reported	[135]
16 patients with an oral administration for 3 months.	Berberine	Patients with UC in clinical remission for at least 3 months.	Anti-inflammatory effects in colonic tissue.	↓ Pro-inflammatory cytokines	Not reported	[136]
46 patients for 12 weeks.	Ginger	active mild to moderate UC	Score of severity of disease activity significantly improved and increased patients’ quality of life.	↓ MDA serum level	Not reported	[137]

↑: Increase; ↓: Decrease; RSV: resveratrol; UC: Ulcerative Colitis; DAI: Disease Activity Index; SOD: Superoxide Dismutase; MDA: Malondialdehyde; TNF-α: Tumor Necrosis Factor-alpha; CD: Crohn’s Disease; OS: Oxidative Stress; TAC: Total Antioxidant Capacity.

## Data Availability

No new data were created or analyzed in this study. Data sharing is not applicable to this article.

## Abbreviations

The following abbreviations are used in this manuscript:

AA	Ascorbic Acid
ACh	Acetylcholine
CD	Crohn’s Disease
ChAT	Choline Acetyl Transferase
DAI	Disease Activity Index
DIO	Diet Induced Obesity
DM	Diabetes Mellitus
EGCs	Enteric Glial Cells
eNOS	Endothelial NOS
ENS	Enteric Nervous System
GE	Ginger
GFAP	Glial Fibrillary acidic Protein
GIT	Gastrointestinal Tract
GLP-1	Glucagon-Like Peptide 1
GPx	Glutathione Peroxidase
GSH	Reduced glutathione
HFD	High-Fat Diet
IBD	Inflammatory Bowel Disease
ICC-MY	Myenteric interstitial cells of Cajal
II	Intestinal Ischemia
IL-1β	Interleukin-1 Beta
IL-6	Interleukin-6
iNOS	Inducible NOS
LA	Alpha-Lipoic Acid
MDA	Malondialdehyde
NF-κB	Nuclear Factor-KappaB
nNOS	Neuronal NOS
NO	Nitric Oxide
NOS	Nitric Oxide Synthase
OS	Oxidative Stress
PD	Parkinson’s Disease
PGP-n	Protein Gene Product neurons
QE	Quercetin
RNS	Reactive Nitrogen Species
ROS	Reactive Oxygen Species
RSV	Resveratrol
SOD	Superoxide Dismutase
TAC	Total Antioxidant Capacity
TNF-α	Tumor Necrosis Factor-alpha
UC	Ulcerative Colitis
VIP	Vasoactive Intestinal Peptide
